# Supplementary material for: Quality of antenatal care and outcomes of Hypertensive Disorders in Pregnancy among antenatal attendees: A comparison of urban and periurban health facilities in Ghana
Source: PLoS One. 2023 Dec 1;18(12):e0294327. doi: 10.1371/journal.pone.0294327 (PMC10691682; doi:10.1371/journal.pone.0294327)
Supplement: S1 Checklist — (DOCX) [file pone.0294327.s001.docx]

STROBE Statement—checklist of items that should be included in reports of observational studies

|  | Item No. | Recommendation | Page  No. | Relevant text from manuscript |
| --- | --- | --- | --- | --- |
| **Title and abstract** | 1 | (*a*) Indicate the study’s design with a commonly used term in the title or the abstract | 2 | cross-sectional study |
|  |  | (*b*) Provide in the abstract an informative and balanced summary of what was done and what was found | 2 | Data on demographics, proportions of HDPs, level of knowledge of mothers on HDPs, quality of ANC and the outcomes of HDPs were collected. There is a considerable periurban-urban disparities among the health indices assessed. |
| Introduction | | | |  |
| Background/rationale | 2 | Explain the scientific background and rationale for the investigation being reported | 3 | There is evidence of health status disparities between the rural and urban women in the Ashanti region with the rural women having worse health outcomes compared to the urban women. However, there is paucity of data of health outcomes of HDPs within the periurban, perhaps their health outcomes maybe better than the rural settlements due to their proximity to the urban areas. |
| Objectives | 3 | State specific objectives, including any prespecified hypotheses | 3 | This study sought to assess the disparities in health indices in the urban-peri urban health facilities concerning hypertensive disorders among pregnant women outcomes in the Ashanti region so as to help promote targeted interventions to improve HDPs outcomes in pregnancy. |
| Methods | | | |  |
| Study design | 4 | Present key elements of study design early in the paper | 4 | It employed a pretested structured questionnaire to obtain data from women who were seeking care at the health care delivery system in the Ashanti region. Antenatal records were also reviewed for other information. |
| Setting | 5 | Describe the setting, locations, and relevant dates, including periods of recruitment, exposure, follow-up, and data collection | 4.5 | Health facilities (hospitals) were located in four different districts of the Ashanti Region. Specifically, two from the urban settings and the other two from the periurban settlements. All data were collected from January to April 2022. |
| Participants | 6 | (*a*) *Cohort study*—Give the eligibility criteria, and the sources and methods of selection of participants. Describe methods of follow-up  *Case-control study*—Give the eligibility criteria, and the sources and methods of case ascertainment and control selection. Give the rationale for the choice of cases and controls  *Cross-sectional study*—Give the eligibility criteria, and the sources and methods of selection of participants | 4 | This study included pregnant women aged 18years and above at the point of delivery. Pregnant women who were not due for delivery and those with co-morbid conditions were excluded. |
|  |  | (*b*) *Cohort study*—For matched studies, give matching criteria and number of exposed and unexposed  *Case-control study*—For matched studies, give matching criteria and the number of controls per case |  | N/A |
| Variables | 7 | Clearly define all outcomes, exposures, predictors, potential confounders, and effect modifiers. Give diagnostic criteria, if applicable |  | N/A |
| Data sources/ measurement | 8* | For each variable of interest, give sources of data and details of methods of assessment (measurement). Describe comparability of assessment methods if there is more than one group | *5* | Their antenatal records were also reviewed for other information. Cronbach alpha test was used to assess the reliability of the construct of composite variables such as the level of knowledge. The quality of ANC was assessed based on the WHO quality indices for ANC 2018. The dependent variable, HDPs, was measured as a mothers having BP above 140/90mmHg beyond 16weeks gestation with or without history of hypertension or evidence of organ damage as confirmed and documented by the midwives or a doctor in the patient’s records book. |
| Bias | 9 | Describe any efforts to address potential sources of bias | 4,5 | A simple random sampling technique was used to select participants and research team was thoroughly trained on the administration of the questionnaire so there was consistency in the data collected.  The study was subject to participant recall bias as participants were made to recall past events. To reduce this limitation, investigators tried to reduce the number of recall items on the questionnaire without compromising on the validity of the study. Also, participants who were not able to readily recall were not forced to provide a response. |
| Study size | 10 | Explain how the study size was arrived at | 4 | Using the Yamane’s formula at a significance level of 5%, an estimated sample size of 440 pregnant women was adequate. Making allowance for non-response rate, a minimum of 500 pregnant women were recruited. |

Continued on next page

| Quantitative variables | 11 | Explain how quantitative variables were handled in the analyses. If applicable, describe which groupings were chosen and why | 6 | Data on the Excel were cross-checked, cleaned, coded, and were imported into Stata 14.1 |
| --- | --- | --- | --- | --- |
| Statistical methods | 12 | (*a*) Describe all statistical methods, including those used to control for confounding | 6 | A univariate analysis employing the Pearson’s Chi square test and univariable logistic regression analysis was carried out to assess the factors associated with HDP. The univariate and multivariable analysis were expressed as crude and adjusted Odds ratio with their respective 95% Confidence Intervals (CIs). Statistical significance was considered at p <0.05. Multiple logistic regression was used to adjust for the effect of known confounders. However, for unknown confounders may be limitations to the study. |
|  |  | (*b*) Describe any methods used to examine subgroups and interactions |  | N/A |
|  |  | (*c*) Explain how missing data were addressed |  | N/A |
|  |  | (*d*) *Cohort study*—If applicable, explain how loss to follow-up was addressed  *Case-control study*—If applicable, explain how matching of cases and controls was addressed  *Cross-sectional study*—If applicable, describe analytical methods taking account of sampling strategy |  | N/A |
|  |  | (*e*) Describe any sensitivity analyses |  | N/A |
| Results | | | | |
| Participants | 13* | (a) Report numbers of individuals at each stage of study—eg numbers potentially eligible, examined for eligibility, confirmed eligible, included in the study, completing follow-up, and analysed | 6 | A total of 500 women were included in this study. |
|  |  | (b) Give reasons for non-participation at each stage |  | N/A |
|  |  | (c) Consider use of a flow diagram |  | N/A |
| Descriptive data | 14* | (a) Give characteristics of study participants (eg demographic, clinical, social) and information on exposures and potential confounders | 6 | The age group, ethnicity, education, religion, occupation, level of knowledge on HDPs by mothers, hypertension status and delivery outcomes were significantly different for women from peri urban and urban facilities. |
|  |  | (b) Indicate number of participants with missing data for each variable of interest |  | N/A |
|  |  | (c) *Cohort study*—Summarise follow-up time (eg, average and total amount) |  | N/A |
| Outcome data | 15* | *Cohort study*—Report numbers of outcome events or summary measures over time |  | *N/A* |
|  |  | *Case-control study—*Report numbers in each exposure category, or summary measures of exposure |  | *N/A* |
|  |  | *Cross-sectional study—*Report numbers of outcome events or summary measures | *8* | Health outcomes for women from urban and peri-urban health facilities differed significantly with regards to the hypertensive state, type of HDPs, quality of ANC, gestational age at delivery, and knowledge of HDPs among the women. The pregnancy and delivery outcomes did not differ significantly between the two groups. |
| Main results | 16 | (*a*) Give unadjusted estimates and, if applicable, confounder-adjusted estimates and their precision (eg, 95% confidence interval). Make clear which confounders were adjusted for and why they were included |  | N/A |
|  |  | (*b*) Report category boundaries when continuous variables were categorized |  | N/A |
|  |  | (*c*) If relevant, consider translating estimates of relative risk into absolute risk for a meaningful time period |  | N/A |

Continued on next page

| Other analyses | 17 | Report other analyses done—eg analyses of subgroups and interactions, and sensitivity analyses |  | N/A |
| --- | --- | --- | --- | --- |
| Discussion | | | | |
| Key results | 18 | Summarise key results with reference to study objectives | 12 | There was a considerable periurban-urban disparities amongst health facilities in Ashanti region. The burden of HDPs was very high in urban health facilities with high proportion of mothers attending and receiving quality ANC care in the urban facilities compared to periurban health facilities. Women in the urban setting also showed excellent knowledge on HDPs as well as better pregnancy outcomes compared to those in the periurban settings |
| Limitations | 19 | Discuss limitations of the study, taking into account sources of potential bias or imprecision. Discuss both direction and magnitude of any potential bias |  | With respect to the recall bias, the magnitude of the effect of the bias cannot be assessed in this study. By virtue of the study design effects of unknown confounders could not be accounted for. |
| Interpretation | 20 | Give a cautious overall interpretation of results considering objectives, limitations, multiplicity of analyses, results from similar studies, and other relevant evidence |  | The burden of HDPs is high in urban settings of the region. However, since it is a cross-sectional study, it is only suitable at providing a snapshot estimate of the burden of HDPs in the region.  Mothers in the urban setting demonstrated high level of knowledge on HDPs as well as being given quality ANC services and hence having better pregnancy outcomes compared to those in the periurban setting. This demonstrates the health disparity between the urban and periurban in the Ashanti region and the need to bridge these gaps. |
| Generalisability | 21 | Discuss the generalisability (external validity) of the study results |  | This study can be generalised to all pregnant women in Ghana |
| Other information | |  | | |
| Funding | 22 | Give the source of funding and the role of the funders for the present study and, if applicable, for the original study on which the present article is based | 12 | Authors received local scholarship from the GNPC Foundation, Ghana. However, funders played no role in the research. |

*Give information separately for cases and controls in case-control studies and, if applicable, for exposed and unexposed groups in cohort and cross-sectional studies.

**Note:** An Explanation and Elaboration article discusses each checklist item and gives methodological background and published examples of transparent reporting. The STROBE checklist is best used in conjunction with this article (freely available on the Web sites of PLoS Medicine at http://www.plosmedicine.org/, Annals of Internal Medicine at http://www.annals.org/, and Epidemiology at http://www.epidem.com/). Information on the STROBE Initiative is available at www.strobe-statement.org.
