# Supplementary material for: Quality of antenatal care and outcomes of Hypertensive Disorders in Pregnancy among antenatal attendees: A comparison of urban and periurban health facilities in Ghana
Source: PLoS One. 2023 Dec 1;18(12):e0294327. doi: 10.1371/journal.pone.0294327 (PMC10691682; doi:10.1371/journal.pone.0294327)
Supplement: Attachment — Submitted filename: Comment-Manuscript-Ploseone.docx [file pone.0294327.s002.docx]

**Manuscript ID:** PONE-D-23-07721

Title: Quality of Antenatal Care (ANC) and Outcomes of Hypertensive Disorders in
Pregnancy (HDPs) among Antenatal Attendees: A Comparison of Urban and Periurban Health Facilities in Ghana

Date: 7 May 2023

**General comment**

This topic of the study, which aimed to compare the difference ANC quality and outcome of HDP among ANC attendees and the sample size is also fine, sounds good. The manuscript was also technically sound, and the data supported the conclusions.

**Comments for the specific area of improvement**

In the abstract: Method: Line 25: it says...... involving 500 (54%, n=270) urban and
26 (46%, n=230) periurban pregnant women was conducted. Why the sample size differ among the areas (Periurban and urban)

In the introduction line 53: it says various studies and cited by only one citation? Authors should address this

Line 54: This is relevant to policy as rural and peri urban areas including health facilities have worse health outcomes compared to urban areas (Zeng et al., 2015). If so, what is the important of your study currently, meant you knew that as health outcome in rural and periurban is worse than urban. Revise it

The same to line 63, this statement make your study insignificant, unless remove it.

Line 67 -68: Perhaps their health outcomes may be better than the rural settlements due to their proximity to the urban areas. This is seems copy paste from a literature and it is implication of other study results. Unnecessary

Method

Study design and site

Line 83-90: you were compared study participants from periurban and urban one: but how could you control the overwhelming of the advanced hospitals over the health facilities found at periurban; meant if women referred to the advanced hospital from periurban or choosing advanced hospital for advanced care by their selves? Any mechanism to oversee such problem??

Sample and sampling

Line 93: Why you had selected women aged 18 and above? Any reason behind? Why not 15 years old?

Line 95: how it come 440 and then 500?? Show with steps?? How much is the non-response rate?

Line 115-117: what is your reference to measure knowledge with this cut points?? Is it appropriate to measure knowledge with such cut points?? Knowledge is continuous variable. It needs explanations

Line 174: table2: it says quality ANC ===poor and good; what is your reference for?

Discussion

The discussion is shallow in general

Line 190-193=== this statement controversial to your topic, it makes incomparable, as stated in the study design and site

Line 251-253: scientifically not sound, meant you compared with figures not statically value

Acknowledgement

Line 292: is the supervisor part of the authorship or not? If he is part of the authorship, no need of special acknowledgement

Generally I accepted this manuscript with minor revision.

Thanks
