# Supplementary material for: Quality of antenatal care and outcomes of Hypertensive Disorders in Pregnancy among antenatal attendees: A comparison of urban and periurban health facilities in Ghana
Source: PLoS One. 2023 Dec 1;18(12):e0294327. doi: 10.1371/journal.pone.0294327 (PMC10691682; doi:10.1371/journal.pone.0294327)
Supplement: Attachment — Submitted filename: Response to Reviewers.docx [file pone.0294327.s003.docx]

Dear Editor,

Thank you for giving us the opportunity to submit a revised draft of the manuscript. We appreciate the time and effort that you and the reviewers have dedicated to providing your valuable feedback on this manuscript. We are grateful to the reviewers for their insightful comments on the paper. We have been able to incorporate changes to reflect most of the suggestions provided by the reviewers. Here is a table showing responses to the reviewers’ comments and concerns.

Reviewer 1

| COMMENT | RESPONSE |
| --- | --- |
| ABSTRACT | |
| The results, as stated in the abstract, needed to provide more data. It reads like a discussion. I suggest the authors report the data to support the statements. | The results section of the abstract has been modified accordingly. |
| HPDs abbreviation in the abstract was not correctly placed. | This has been rectified |
| Please proofread the manuscript. | The manuscript has been proofread for errors |
| INTRODUCTION | |
| Lines 50 to 51: Provide references. | This has been rectified. Adequate references have been provided |
| Lines 60 to 61: The references are more than 10 years and cannot be the current evidence. | This reference has been replaced. |
| Please be consistent with HDP usage throughout the manuscript. Sometimes Hypertensive disorders or hypertensive disorders of pregnancy is used. | The manuscript has been reviewed to ensure that all HDPs abbreviations are consistent. |
| When a region is used, indicate the country. | This has now been indicated throughout the manuscript. |
| The aim stated in the abstract, and the introduction are not consistent. This made it difficult to review the manuscript. | Although both paragraphs meant the same, they have been synchronized to provide better clarity. |
| METHOD | |
| 18 years and above was the only inclusion criteria? Did the participants receive any incentives? | This study included pregnant women above the ages of 18years who had been admitted for delivery and those who had delivered at the hospital during the period of the study.  Participants were not given any incentives. |
| Both independent and dependent variables were not clearly defined. Which diagnoses were included in HDP? Chronic hypertension, gestational hypertension, preeclampsia? | The dependent variable was the location of the health facility, that is either urban or periurban health facility.  The independent variables were the quality of ANC, outcome of pregnancy, type of HDPs and knowledge of mothers on HDPs  For this study, HDPs included Chronic hypertension, chronic superimposed with preeclampsia, gestational hypertension and  Pre-eclampsia.  Diagnosis was done using the Ghana Standard Treatment Guidelines (STG 2017) and the American College Of  Obstetricians and Gynaecologists guidelines 222. |
| Hypertension before 20 weeks gestation is considered pre-existing/chronic hypertension. It is not clear the criteria that support “blood pressure above 140/90 beyond 16 weeks gestation” as HDP. Clearly defining the variables could help. | This has been rectified accordingly |
| The data analysis section needed to be revised. | The section has been revised accordingly. |
| RESULTS AND DISCUSSION | |
| Due to missing information on the method, and inconsistent objectives, I could not evaluate the results and discussion adequately. |  |

Reviewer 2

| COMMENT | RESPONSE |
| --- | --- |
| ABSTRACT | |
| In the abstract: Method: Line 25: it says...... involving 500 (54%, n=270) urban and 26 (46%, n=230) periurban pregnant women was conducted. Why the sample size differ among the areas (Periurban and urban) | This manuscript forms a part of cohort study that was conducted. As such the categories urban and periurban were not the outcome of interest for the conduct of the study. Therefore, these categories did not influence the sample size determination nor the sampling approach.  However, analysis of the study revealed interesting findings of health outcome disparities between the urban and periurban participants which the authors of this manuscript believe would contribute to the knowledge of high HDPs in Ghana. |
| INTRODUCTION | |
| In the introduction line 53: it says various studies and cited by only one citation? Authors should address this. | This has been rectified |
| Line 54: This is relevant to policy as rural and peri urban areas including health facilities have worse health outcomes compared to urban areas (Zeng et al., 2015). If so, what is the important of your study currently, meant you knew that as health outcome in rural and periurban is worse than urban. Revise it | This line has been revised |
| The same to line 63, this statement make your study insignificant, unless remove it. | This sentence has removed. |
| Line 67 -68: Perhaps their health outcomes may be better than the rural settlements due to their proximity to the urban areas. This is seems copy paste from a literature and it is implication of other study results. Unnecessary | This sentence has been removed |
| METHOD | |
| Study design and site |  |
| Line 83-90: you were compared study participants from periurban and urban one: but how could you control the overwhelming of the advanced hospitals over the health facilities found at periurban; meant if women referred to the advanced hospital from periurban or choosing advanced hospital for advanced care by their selves? Any mechanism to oversee such problem?? | For the cohort study, participants consented to part of the study agreed to visit and deliver at the specified hospital during the duration of study.  As per the protocol, participants who were referred for another hospital or relocated from the catchment area during the conduct of the study were considered to have deviated or lost to follow up. Such participants are not included in the analysis. Therefore, participants who were part of this study continued with their visits and delivered at the specific health facility during the study period. |
| Sample and sampling |  |
| Line 93: Why you had selected women aged 18 and above? Any reason behind? Why not 15 years old? | In Ghana, an 18-year-old is legally considered a full-fledged adult with rights and responsibilities.  Therefore, such individuals (18years and above) can decide for themselves whether to agree to participate in the study or not.  For those younger than 18years, consent to participate in the study must be sought from their parents or guidance, which might cause a bit of delay. |
| Line 95: how it come 440 and then 500?? Show with steps?? How much is the non-response rate? | The addition of 60participants was to take care of possible non-response from participants from the four facilities which could affect the external validity of the sample. |
| Line 115-117: what is your reference to measure knowledge with this cut points?? Is it appropriate to measure knowledge with such cut points?? Knowledge is continuous variable. It needs explanations | There were no references to the measure of knowledge as the items for assessing knowledge were authors own construct after several literature review and pretesting of the measurement tool. |
| Line 174: table2: it says quality ANC ===poor and good; what is your reference for? | There were no references to the measure of ANC. However, the measurements were done in line with the WHO requirements for ANC quality indices. This was used to obtain a score and participants who scored more than half of the total were considered to have had good ANC. |
| DISCUSSION | |
| The discussion is shallow in general | This has been improved. |
| Line 190-193=== this statement controversial to your topic, it makes incomparable, as stated in the study design and site | This statement has been revised. |
| Line 251-253: scientifically not sound, meant you compared with figures not statically value | This has been revised |
| ACKNOWLEDGEMENT | |
| Line 292: is the supervisor part of the authorship or not? If he is part of the authorship, no need of special acknowledgement | This has been rectified. |

We look forward to hearing from you in due time regarding our submission and to respond to any further comments you may have.
